# Categorizing Two Taiwanese Major Political Parties From Their Faces: The Influence of Provincial Appearance

**DOI:** 10.3389/fpsyg.2018.00271

**Published:** 2018-03-21

**Authors:** Chien-Kai Chang, Mary Wen-Reng Ho, Sarina Hui-Lin Chien

**Affiliations:** ^1^Graduate Institute of Neural and Cognitive Sciences, China Medical University, Taichung, Taiwan; ^2^Graduate Institute of Biomedical Sciences, China Medical University, Taichung, Taiwan

**Keywords:** face-to-trait inference, political membership, Kuomintang vs. Democratic Progressive Party, provincial appearance, mainlanders vs. native Taiwanese, social perception

## Abstract

People go beyond the inferences afforded by a person’s observable features to make guesses about personality traits or even social memberships such as political affiliations. The present study extended Hu et al. (2016) to further investigate the influence of provincial appearance on differentiating KMT (Kuomintang) and DPP (Democratic Progressive Party) candidates by headshot photos with three experiments. In Experiment 1 (Membership categorization task), participants categorized the photos from the pilot study (where the difference between the perceived age of KMT and DPP candidates was reduced) and divided into four blocks by their perceived age. We found that participants were able to distinguish KMT from DPP candidates significantly better than chance, even when the perceived age difference between the two parties was minimized. In Experiment 2 (Trait rating task), we asked young and middle-aged adults to rate six traits on candidate’s photos. We found that “provincial appearance” is the core trait differentiating the two parties for both young and older participants, while “facial maturity” is another trait for older participants only. In Experiment 3 (Double categorization task), we asked participants to categorize the photos from the Exp. 1 on their membership (KMT or DPP) and on provincial appearance (mainlander or native Taiwanese) in two separate sessions. Results showed that young adults were likely to use the “provincial appearance” as the main characteristic cue to categorize candidates’ political membership. In sum, our study showed that Taiwanese adults could categorize the two parties by their headshot photos when age cue was eliminated. Moreover, provincial appearance was the most critical trait for differentiating between KMT and DPP candidates, which may reflect a piece of significant history during the development of the two parties.

## Introduction

Humans are remarkable face recognition experts. In a single glance, one can easily recognize a person’s observable features such as identity, age, sex, or emotions with relatively little information (Gosselin and Schyns, 2001, 2004). Moreover, accuracy on recognizing these facial attributes is not only robust but also tolerant of a variety of image degradations (Sinha et al., 2006). On the other hand, personality traits, such as shyness, competence, extroversion-introversion, or dominance are not apparent features; nevertheless, people are capable of inferring them from facial appearances with rather impressive speed and accuracy (for recent reviews, see Tskhay and Rule, 2013; Todorov et al., 2015). The study of how the appearance of human face correlates with personal characteristics, or the so-called “face-to-trait inference,” can be traced back to the ancient Greek philosophers, as they judged prospective students by how gifted they looked at the admission interview (Waldorf, 2012). Likewise in contemporary elections, from the moment voters glanced at candidates’ faces, they automatically categorize them into their in-group or out-group; in another word, voters affiliate themselves to candidates that they can closely relate to.

Facial appearance affects our social judgments profoundly (Macrae and Bodenhausen, 2000; Zebrowitz and Montepare, 2008; Todorov et al., 2013). The face-to-trait inference not only occurs extremely fast (<50 ms, Willis and Todorov, 2006; Todorov et al., 2009), it also shows broad and cross-cultural consensus with the attributed traits (Rule et al., 2010a), and some aspects may have an early ontogeny in childhood (Antonakis and Dalgas, 2009; Cogsdill et al., 2014; Cogsdill and Banaji, 2015). Moreover, such inference is not limited to personality traits; people can even guess membership of perceptually ambiguous social groups with better-than-chance performance (Rule et al., 2009; Tskhay and Rule, 2013), such as religion (Rule et al., 2010b) and political affiliation (Rule and Ambady, 2010), which is the primary focus of the present study.

In North America, Rule and Ambady (2010) demonstrated that university students could categorize Democrats and Republicans by looking at their headshot photos, regardless of the candidates’ age or experience. Moreover, such categorization was achieved via spontaneous trait inference, where the faces of Democrats were perceived to be warmer (likeability, trustworthiness), and the faces of Republicans were perceived to be more powerful (dominance and facial maturity). In a follow-up cross-cultural study with participants from Japan and United States, Rule et al. (2010a) reported that American participants showed candidates rated high on “power” would be more likely to get elected in the United States, whereas Japanese participants showed candidates rated high on the “warmth” would be more likely to get elected in Japan. In Europe, Samochowiec et al. (2010) indicated that university students could differentiate the right wing and left wing politicians by their photos at a higher-than-chance level. Franklin and Zebrowitz (2016) reported an age effect of the participants; young adults and older adults tended to focus on different traits from candidates’ photos, and such differences form the basis of the predicted election outcomes. In a recent study, Berggren et al. (2017) showed voters from Europe, North America, and Australia used facial attractiveness as a cue for candidate ideology, and beauty has an advantage in elections.

Taiwan, like many other democratic countries, is a nation practicing a multi-party system. However, the two dominant parties, the Democratic Progressive Party (DPP) and the Kuomintang Party (KMT), are vastly known by the citizens and in the East Asian region. The DPP party is known as liberalism because of their strong support for human rights and environmental issues, and the KMT party is known as a republicanism party who once was the only ruling party in Taiwan until the late 1980s. In a recent study from our laboratory, Hu et al. (2016) demonstrated that Taiwanese young adults and mid-age adults could categorize KMT and DPP with accuracies higher than chance by looking at the candidates’ full headshot photos. Moreover, this categorization ability was not jeopardized by reducing information from the exterior contour or the mouth-and-chin area of the face, nor enhanced with voting experience. Hu et al.’s (2016) demonstrated the cross-cultural generality of Rule and Ambady’s (2010) results. Nevertheless, two limitations must bear in mind. First, there was a small perceived age difference in candidates’ photos from KMT and DPP, and such difference can be a confounding factor in the judgment of political affiliations, if participants use age as a cue (i.e., KMT are older than DPP). Second, in contrast to Rule and Ambady (2010), the typically perceived traits for KMT and DPP were unexplored in Hu et al. (2016). Thus we wonder whether dominance, facial maturity, likeability, trustworthiness, youthfulness, and provincial appearance may be important traits to differentiate between the KMT and DPP. The particular trait “provincial appearance” was based on the status of Taiwanese. The term “mainlanders or extraprovincial” (waishengren) were used to describe people who moved to Taiwan from mainland China after 1949, while the term “native Taiwanese (benshengren)” are usually referred to those that lived in Taiwan before 1949. The Mainlanders who fled from China were mainly KMT troops; the KMT party ruled Taiwan from 1945 and remained a single-party state until late 1980s. It is since then a traditional distinction between Waishengren (extraprovincial) and Benshengren (native Taiwanese) (Corcuff, 2002) influenced the political ideology in Taiwan.

The present study extended Hu et al. (2016) to explore further the effect of age, and the traits on differentiating KMT and DPP candidates from their photos with one pilot study and three experiments. The pilot study consisted of a paper-and-pencil perceived age rating task, where the participants rated the candidates’ age based on their headshot photos. We explored the perceived age of KMT and DPP candidates’ photos and aimed to reduce the difference. Experiment 1 was a computerized political membership categorization task, where participants categorized the adjusted photo set from the pilot study. To further minimize the possible bias of the perceived age of candidates’ photos on categorization; we adopted a block design where the candidates’ photos were divided into four blocks by their mean perceived age range. Experiment 2 was a paper-and-pencil trait rating task, exploring which trait was critical for differentiating between KMT and DPP. The participants were given six target traits (dominance, facial maturity, likeability, trustworthiness, youthfulness, and provincial appearance) to rate the candidates from the scale of 1 (least likely) to 7 (very likely). Lastly, Experiment 3 directly tested the link between political membership and provincial appearance. The participants were asked to categorize the photos from the Experiment 1 on their political affiliation as well as whether they resemble mainlander or native Taiwanese.

## Materials and Methods

### Pilot: Perceived Age Rating Task

#### Participants

In the pilot study, we recruited a total of 24 participants (12 females) with a mean age of 23.66 years old (*SD* = 3.13 years). The sample size was determined before data analysis and the number of participants was chosen to comply with the study from Hu et al. (2016). The participants were informed of the general purpose and their written consent was obtained before the rating task. All participants self-reported with a normal or corrected-to-normal vision (20/20). All participants reported having no affiliation with any political parties and were naïve to the purpose of the study. After finishing the pencil-and-paper questionnaire, they received a cash payment for their participation. All participants retained in the final data set (*N* = 24). The present study adhered to the guidelines suggested by the Institution Review Board of China Medical University Hospital Research Ethics Committee, Taichung, Taiwan.

#### Stimuli and Procedures

The stimuli consisted of 100 gray-scale photos of current KMT and DPP members (50 photos each) and were accessed from the website of 2012 Taiwanese Legislative Election by the Central Election Commission^1^. The ratio of male and female candidates was set to 4 to 1 (40 male photos and ten female photos from each party). This ratio of 4 to 1 was close to the base rates of the population from both parties. Photos of candidates with high media exposure were not included; the number of candidates with glasses in both parties was approximately equal. We used pencil-and-paper questionnaire which had four A4 pages, and each page contained 25 photos of the selected candidates displayed in five rows. Each photo was numbered and sized to 3.3 cm (height) by 3.0 cm (width). Participants were asked to rate the perceived ages of the 100 candidates’ photos into the five given age ranges, 20–29, 30–39, 40–49, 50–59, and 60–69 years old, on a separate answer sheet.

#### Results

First, we encoded the participant’s answers of the perceived age ranges with the median of the age intervals. For example, if a participant rated the perceived age of a particular candidate in the age interval of 30–39 years old, the perceived age would be 34.5 years old. The mean perceived age of individual candidate’s photo was obtained by averaging across all of the participants’ correspondent answers. For instance, if participant A rated the perceived age of Candidate X in the age interval of 30–39 age interval (median: 34.5) and participant B rated the perceived age of same candidate in the age interval of 40–49 age interval (median: 44.5), the mean perceived age of Candidate X for participants A and B will be 39.5 years old.

A three-way mixed ANOVA was conducted to examine if there is a difference in perceived age between KMT and DPP candidates’ photos. We mainly focused on the main effects. The between-subject factor was Participants’ Gender (males vs. females), the within-subject factors were Party (KMT vs. DPP) and Photo Gender (male vs. female photos). The main effect of Party was significant; the mean perceived age of the KMT’s photos (*M* = 46.41 years old, *SD* = 6.52 years) was significantly higher than that of the DPP’s photos (*M* = 44.26 years old, *SD* = 7.46 years), *F*(1,22) = 89.670, *p <* 0.001, η^2^ = 0.803. The main effect of Photo Gender was also significant; the mean perceive age of male candidates’ photos (*M* = 46.74 years old, *SD* = 4.41 years) was significantly higher than that of female photos (*M* = 39.69 years old, *SD* = 4.08 years), *F*(1,22) = 267.192, *p <* 0.001, η^2^ = 0.924. The main effect of Participant’s Gender was not significant (*p* = 0.522).

**FIGURE 1:**
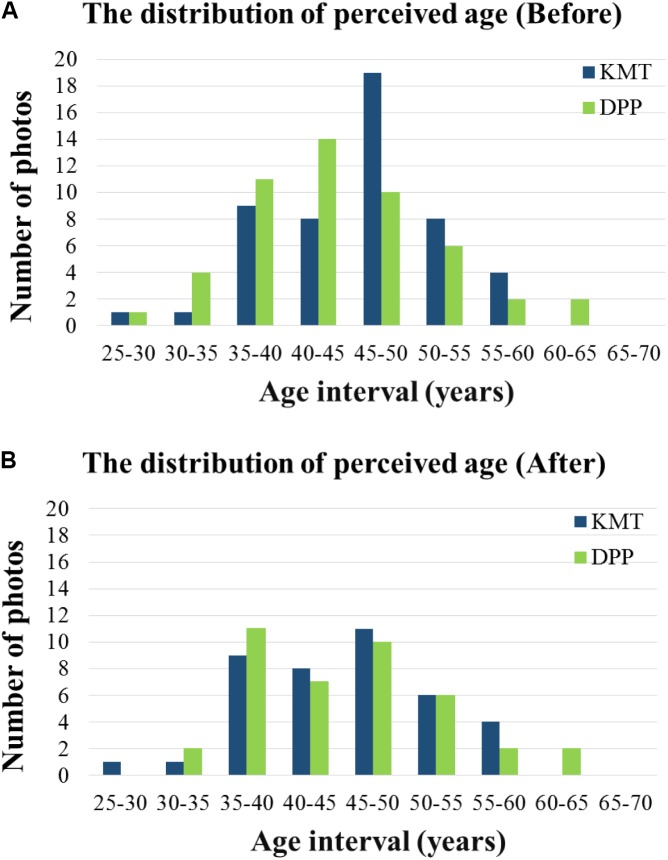
The distribution of KMT and DPP candidates’ photos in the pilot study before **(A)** and after **(B)** the adjustment. The abscissa denotes the perceived age interval (in 5 years increment), and the ordinate represents the numbers of photos. The blue bars represent KMT candidates, and the green bars represent DPP candidates.

To give a clear view of the distribution of perceived age for both parties, we plotted the frequency charts of the KMT candidates and the DPP candidates using 5% bins. As shown in **Figure 1A**, the distribution of perceived age of KMT candidates largely overlapped with the distribution of the DPP candidates. However, the KMT candidates’ peak frequency of the perceived age fell within the 45–50 age interval, while the DPP candidates’ peak frequency of the perceived age fell within the 40–45 age interval. In summary, we discovered that the mean perceived age of KMT candidates’ photos was significantly higher than that of DPP candidates by about 2 years, and the perceived ages of male candidates were significantly higher than that of female candidates by 7 years.

#### Adjusting the Perceived Age

To prepare for Experiment 1 (Membership categorization task), we minimized the perceived age differences between KMT and DPP candidates’ photos by excluding ten photos from each party. As shown in **Figure 1A**, the major difference in the frequency distribution of the perceived age centered in the 40–45 and 45–50 age intervals; thus we focused on adjusting the candidates’ photos in these two age intervals. For the KMT candidates, we excluded six male photos from the 45–50 age interval, two male photos from the 50–55 age interval, and two oldest female photos from the 50–55 age interval. For the DPP candidates, we excluded one male and one female photo from the 30–35 age interval, seven male photos from the 40–45 age interval, and one female photo from the youngest 25–30 age interval. After the adjustment, a total of 80 candidates’ photos retained, 40 from each party. The adjusted mean perceived ages for KMT and DPP were 45.63 years old (*SD* = 7.06 years) and 45.59 years old (*SD* = 7.37 years), respectively, and the difference was not significant [*t*(39) = 0.027, *p* = 0.979]. Moreover, for male candidates, the mean perceived ages between KMT (*M* = 46.88 years old, *SD* = 7.37 years) and DPP (*M* = 47.26 years old, *SD* = 7.13 years) were not significant [*t*(31) = -0.200, *p* = 0.843]. For female candidates, the mean perceived age between KMT (*M* = 40.65 years old, *SD* = 5.84 years) and DPP (*M* = 38.93 years old, *SD* = 3.82 years) were also not significant [*t*(7) = 0.739, *p* = 0.484]. **Figure 1B** illustrates the distribution of the remaining 80 candidates’ perceived age. All measures, manipulations, and exclusions in the pilot study were reported.

## Experiment 1: Political Membership Categorization Task

Experiment 1 aimed to examine whether Taiwanese under graduate students can categorize KMT or DPP candidates from their photos. We adopted the remaining 80 photos from the pilot study and separated them into four blocks by their perceived age range to further minimize the effect of perceived age on categorization bias. The participants’ task was to categorize 80 photos of candidates as either DPP or KMT members presented in four blocks.

### Participants

In Experiment 1, we recruited a total of 40 (20 females) non-politically affiliated undergraduates. The participants’ age was between 18 and 29. The sample size was determined before any data analysis, and the number of participants was chosen to comply with the study from Hu et al. (2016; Experiment 1 *N* = 35 participants). They were primarily students at China Medical University, Taichung, Taiwan. Written informed consent was obtained before the experiment. All participants self-reported with normal or corrected-to-normal vision (20/20) and were naïve to the purposes of the experiment. Each participant was tested individually in a quiet, moderately lit room. Before taking part in the computerized categorization task, participants will fill out a basic demographic and voting experience questionnaire, including their age, years of education, political affiliation, and the numbers of elections for which they have voted since 2004. After completing the categorization task, the participants received a cash payment for their participation. Two participants were later excluded due to the following reasons; one had previously joined a similar study, the other correctly recognizes a large number of candidates (26 out of the 80 candidates). The final data set consisted of 38 participants (19 females) with an average age of 22.29 years old (*SD* = 2.46 years).

### Apparatus and Stimuli

Experiment 1 was a computerized two-alternative-forced-choice (2FAC) categorization task. A desktop computer (Shuttle SH67H) with 22′′ LCD monitor (Chimei CMV 221) and E-Prime Professional 2.0 (Psychological Software Tools, Sharpsburg, PA, United States) were used to run the experiment. The participants seated on chairs adjusted to their heights such that their eyes could fixate on the center of the screen, at a distance of approximately 57 cm. The stimuli consisted of 80 gray-scale photos of current KMT and DPP members from the pilot study, 40 photos for each party and the ratio of male and female candidates was 4 to 1. Each selected photo was cropped and resized proportionally to 21 cm (width) by 17 cm (height) on the monitor display occupying approximately 21° by 17° in the visual angle at a viewing distance of 57 cm. To control for the possible effect of perceived age on categorization bias, we further divided the 80 candidates’ photos into 4 blocks (20 photos for each block) by the perceived age rating (under 40 years old, 40–45 years old, 45–50 years old, and above 50 years old) from the pilot study. In each block, the numbers of KMT and DPP candidates were equal (10 photos each) and their mean perceived ages were not significantly different (block 1: KMT: 36.92 years old, DPP: 37.79 years old, *p* = 0.513; block 2: KMT: 43.46 years old, DPP: 43.29 years old, *p* = 0.910; block 3: KMT: 48.42 years old, DPP: 47.25 years old, *p* = 0.503; block 4: KMT: 53.75 years old, DPP: 54.04 years old, *p* = 0.904).

### Design and Procedures

**FIGURE 2:**
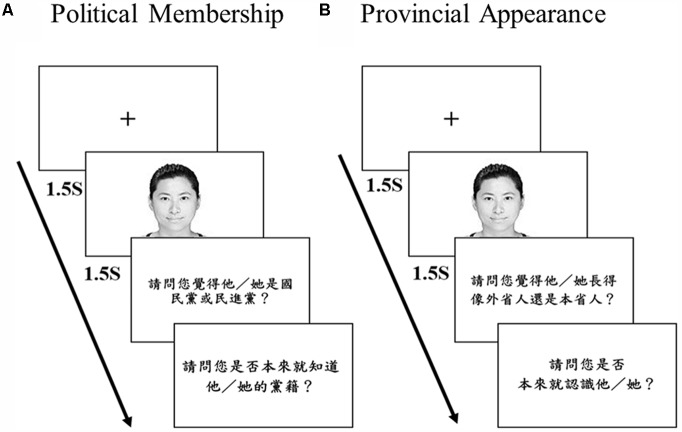
The illustration of a sample trial in the political membership categorization task of Experiments 1 and 3 **(A)**, and a sample trial in the provincial appearance categorization task of Experiment 3 **(B)**. The translation of the first question in **(A)** is “Which political party do you think he/she belongs to, KMT or DPP?” and that of the second question is “Do you already know his/her political party?” The translation of the first question in **(B)** is “Which provincial group do you think he/she belongs to, Mainlander or Native Taiwanese?” and that of the second question is “Do you already know this person?”

Participants were asked to categorize 80 photos of candidates as either DPP or KMT members presented in four blocks. The test order of the four blocks was counter-balanced among participants. Within each block, the 20 photos were presented in random order. **Figure 2A** illustrates a sample trial of the computerized membership categorization task. Each trial began with a fixation cross for 1.5 s, followed by a photo of either a DPP or a KMT candidate presented for 1.5 s, and the participants were prompted with two subsequent questions without time limit: “Which political party do you think he/she belongs to, KMT or DPP?” and “Do you already know his/her political party?” When participants answered the first question, the second question appeared on the screen and remained on until the participants made a yes/no response. Then the next trial began. Before the four blocks of formal categorization task, the participants received a practice block consisting 20 famous political candidates (10 for each party) to ensure that they familiarized themselves with the procedure of the categorization task. After completing the practice block and the four blocks of formal trials, each participant was interviewed by the experimenter with an open question “How did you guess?” The mean accuracy of practice block was 0.916 (*SD* = 0.080), suggesting that the participants understood the task well and could recognize most of the photos in the practice block. For the experimental trials, those trials that the participants reported to have known the political party of the candidates were excluded in the subsequent analyses. The average number of excluded trials was 3.55 and the median was 4.

### Results

The primary goal of Experiment 1 was to examine whether participants could categorize political membership of KMT and DPP candidates by their photos better than chance after the difference in perceived age has been minimized. The accuracy of categorizations of each participant was measured by percentage correct of the items he/she did not indicate recognition. Sensitivity measure *d*’ and response bias *c* were calculated based on the hit rates of and false alarms of KMT items. We compared the categorization accuracy with chance level (0.5) by one-sample *t*-test, the group mean accuracy of categorization task (*M* = 0.530, *SD* = 0.063) was significantly higher than chance, *t*(37) = 2.948, *p* = 0.0028, Cohen’s *d* = 0.969, suggesting that like Hu et al.’s (2016) finding, our young adult participants could categorize KMT and DPP members by their photos when the perceived age difference has been minimized. We also analyzed the sensitivity index d prime (*d’*) and the response bias *c* based on the Signal Detection Theory. The hit rate for KMT was 0.543, the false alarm rate 0.485, yielding a *d’* = 0.160 (*SD* = 0.343). The response bias *c* was -0.037 (*SD* = 0.241), indicating a slight bias toward categorizing photos as DPP. The categorization accuracy between KMT (*M* = 0.544, *SD* = 0.116) and DPP (*M* = 0.515, *SD* = 0.101) was not significantly different (*p* = 0.311).

**FIGURE 3:**
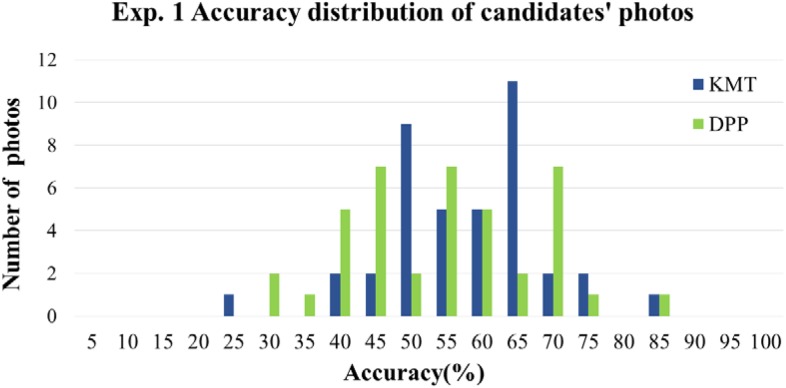
The distribution of item difficulties in Experiment 1, measured by the percentage correct collapsed across participants. The abscissa denotes the categorization accuracy of the candidates’ photos in 5% bins; the ordinate denotes the number of photos.

To ensure that the above results were not due to a few extremely easily categorized photos that biased the overall accuracy upward (or extremely difficult items that pulled the accuracy downward), we further analyzed the distribution of item difficulties. The difficulty of the photos was defined as the correct percentage of each trial. As shown in **Figure 3**, the correct percentage of candidates’ photos were between 25 to 90%, none of the accuracies of the photos reached 100 or 0%, meaning that the aforementioned greater-than-chance performance was not due to just a few photos that could be easily categorized.

Lastly, we also analyzed the correlation between categorization accuracy and each item of the participants’ basic demographic information (age, years of education, and the numbers of elections). None of the results showed significant correlation between categorization accuracy and each item of the participants’ basic information (age: *r* = 0.225, *p* = 0.175; years of education: *r* = 0.164, *p* = 0.326; and the numbers of elections: *r* = 0.121, *p* = 0.471). All measures, manipulations, and exclusions in Experiment 1 were reported.

## Experiment 2: Trait Rating Task

To explore the possible traits allowing the participants to differentiate between KMT and DPP candidates from their photos, we designed a paper-and-pencil trait rating task. The participants were asked to rate six target traits, Dominance, Facial Maturity, Likeability, Trustworthiness, Youthfulness, and Provincial Appearance, with a 7-point rating scale. As Franklin and Zebrowitz (2016) reported that young and older adults tended to focus on different traits when viewing candidates’ photos, thus, we recruited young adults and middle-aged adults to explore the possible effect of participants’ age in rating these candidates’ photos.

### Participants

In Experiment 2, we recruited a total of 55 non-politically affiliated participants. The sample size was determined before any data analysis, and the number of participants was chosen to comply with Rule and Ambady’s (2010; 46 participants). Twenty nine of them (18 females) were assigned to the young adult group, with a mean age of 23.75 years old (*SD* = 2.46 years), while 26 of them (15 females) were assigned to the middle-aged adult group, with a mean age of 47.78 years old (*SD* = 7.32 years). Written informed consent was obtained before the experiment. All participants self-reported with a normal or corrected-to-normal vision (20/20) and were naïve to the purposes of the experiment. They were tested individually in a quiet, moderately lit room. After completing the task, each participant received a cash payment for their participation. All participants were retained in the final data set.

### Stimuli and Procedures

**FIGURE 4:**
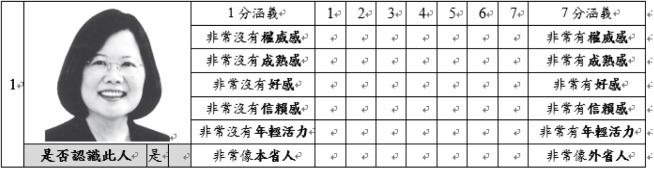
A practice question of the trait rating task questionnaire in Experiment 2. Photo shown is the current Taiwanese president, Ing-Wen Tsai, whose photo was not included in the actual rating task (The photo was obtained from the 2012 Taiwanese President Election by the Central Election Commission website). The question below the candidate’s photo is “Do you recognize this person?”. The table on the right side of the candidate’s photo lists the six-trait rating questions on the 7-point scale (from top to bottom: *Dominance, Facial Maturity, Likeability, Trustworthiness, Youthfulness, and Provincial Appearance*).

Experiment 2 adopted a pencil-and-paper questionnaire similar to the pilot study. In Experiment 2, we divided the 80 photos from the pilot study into two questionnaires, A and B. Each questionnaire contained 40 candidates’ photos, and the ratio of male to female candidates was 4 to 1. The mean perceived age between questionnaires A and B was not different (*p* = 0.979). The participants received either Questionnaire A or Questionnaire B. The questionnaire included 40 items, each item contained one target photo, six traits (Dominance, Facial Maturity, Likeability, Trustworthiness, Youthfulness, and Provincial Appearance) for rating, and one question for photo familiarity. **Figure 4** illustrates an example of an item. For the six target traits, participants were asked to rate the target traits on a 7-point scale. For the first five traits, Dominance, Facial Maturity, Likeability, Trustworthiness, and Youthfulness, the rating scores ranged from “1” being “not a bit” to “7” being “very much” (for example, if a participant rated a candidate’s photo’s Dominance as 1 means that this candidate did not appear to be dominant at all). For the sixth trait “Provincial Appearance,” the rating was different in its nature. The 7-point rating scale of “Provincial Appearance” referred to the judgment of candidates’ appearance between the native Taiwanese (Hoklo and Hakka people who have been in Taiwan prior to the mass exodus near the end of the Chinese Civil War) and mainlanders (people who emigrated from Mainland China near the end of the Chinese Civil War and their descendants). The rating score of “1” indicated that the candidate’s appearance highly resembled the native Taiwanese physiognomy whereas the rating of “7” indicated that the candidate’s appearance highly resembled the mainlanders’ physiognomy). The last question is to check participant’s familiarity about the candidate; an item will be excluded from final analysis if the participant answered “yes” (meaning that the participant already knows this person) for this question. The average number of items being excluded due to familiarity was 2.69 (*SD* = 4.21) for young adults and 7.15 (*SD* = 6.27) for middle-aged adults.

### Results

#### The Trait Rating Scores for KMT and DPP in Young and Middle-Aged Adults

**Table 1** summarizes the group means rating scores for the six traits in young and middle-aged adults. To find the traits that can best differentiate KMT and DPP, we compared the rating scores between the KMT and DPP for each of the six traits with multiple comparisons using paired *t*-tests. To control for α expansion, we used Bonferroni correction and set the level of significance α = 0.05/6 = 0.0083. For the young adults, among the six target traits, the only trait exhibiting a marginally significant difference between KMT and DPP was “provincial appearance.” The mean “provincial appearance” rating score for the KMT candidates’ photos (*M* = 3.697, *SD* = 0.404) was significantly higher than DPP candidates’ photos [*M* = 3.472, *SD* = 0.478, *t*(28) = 2.589, *p* = 0.015, Cohen’s *d* = 0.979]. Even if the young adult participants did not know the membership of the to-be rated candidates in reality, nevertheless, they still perceived the KMT candidate’s appearance as resembling the mainlanders while the DPP candidates’ appearance resembling the native Taiwanese.

**Table 1:** The six trait rating scores in young and middle-aged adults in Experiment 2, and the comparisons between the two age groups.

Traits	Young			Middle-aged			Comparison between age groups (*p*-value)	
	KMT	DPP	*p*-value	KMT	DPP	*p*-value	KMT	DPP
Dominance	4.143	4.197	0.547	4.289	4.170	0.078	0.501	0.885
Facial maturity	4.836	4.758	0.153	4.724	4.518	0.015^∗^	0.481	0.132
Likeability	4.340	4.317	0.747	4.157	4.038	0.127	0.229	0.041^∗^
Trustworthiness	4.375	4.429	0.427	4.154	4.050	0.095	0.161	0.008^∗∗^
Youthfulness	4.140	4.159	0.792	3.973	4.051	0.585	0.32	0.493
Provincial appearance	3.697	3.472	0.015^∗^	3.845	3.301	<0.001^∗∗∗^	0.245	0.267

^∗^*p < 0.05, ^∗∗^*p <* 0.01, ^∗∗∗^*p* < 0.001*.

For the middle-aged adults, we also compared the rating scores between the KMT and DPP for each of the six traits using paired *t-tests* with adjusted α level (α = 0.05/6 = 0.0083). As shown in **Table 1**, like the young adults, the trait provincial appearance exhibited significant differences between KMT and DPP candidates. The mean provincial appearance rating score of the KMT candidates’ photos (*M* = 3.845, *SD* = 0.535) was significantly higher than that of the DPP candidates’ photos [*M* = 3.301, *SD* = 0.648, *t*(25) = 4.227, *p <* 0.001, Cohen’s *d* = 1.691]. In addition, the mean Facial Maturity rating score of the KMT candidates’ photos (*M* = 4.724, *SD* = 0.670) was marginally higher than that of the DPP candidates [*M* = 4.518, *SD* = 0.682, *t*(25) = 2.618, *p* = 0.015, Cohen’s *d* = 1.047]. Even though the participants did not know the actual political affiliation of candidates they rated, the provincial appearance and Facial Maturity rating scores were still higher for KMT candidates’ than DPP candidates. In other words, the middle-aged adults seemed to perceive KMT candidates’ appearance to be more mature and resemble the mainlander while the DPP candidates to be less mature and resemble the native Taiwanese.

To reveal if there were differences in rating KMT and DPP candidates for the six traits between the two age groups, we conducted independent *t*-tests. The rating scores of Likeability and Trustworthiness of the DPP candidates’ photos in young adults (Likeability: *M* = 4.317, *SD* = 0.390; Trustworthiness: *M* = 4.429, *SD* = 0.465) were significantly higher than those of the middle-aged adults [Likeability: *M* = 4.038, *SD* = 0.595; Trustworthiness: *M* = 4.050, *SD* = 0.556. Likeability: *t*(53) = 2.094, *p* = 0.041, Cohen’s *d* = 0.616; Trustworthiness: *t*(53) = 2.756, *p* = 0.008, Cohen’s *d* = 0.818]. This suggested that, on average, the young adults found the appearance of DPP candidates more likable and more trustworthy than the middle-aged adults did.

#### Correlation Between Categorization Accuracy and Trait Rating

To explore if there is an association between the perceived trait and the accuracy on political membership categorization, we further analyzed the correlation between the categorization accuracy of KMT and DPP candidates obtained in Experiment 1 (based on the responses of a different group of participants) and the rating scores of the six traits for young and middle-aged adults. The correlations are shown in **Table 2**. For the young adults, the provincial appearance rating scores (the smaller the number, the more resembling Native Taiwanese) of DPP candidates’ photos exhibited a significant negative correlation with the accuracy of membership categorization of the DPP candidates’ photos (*r* = -0.474, *p* = 0.002). Meanwhile, the Likeability rating score of the KMT candidates showed a significant negative correlation with the accuracy of membership categorization (*r* = -0.368, *p* = 0.019). This suggested that the lower the Likeability of the KMT candidates’ photos, the higher the accuracy in categorizing their political membership as KMT correctly. For the middle-aged adults, however, none of the six traits rating scores of the KMT and DPP candidates exhibited a significant correlation with the accuracy of political membership categorization task from Experiment 1.

**Table 2:** The Pearson’s correlations between young and middle-aged adults’ trait rating scores for KMT and DPP candidate photos in Experiment 2 and the young adults’ political membership categorization accuracy in Experiment 1.

Traits	Young adults		Middle-aged adults	
	KMT	DPP	KMT	DPP
Dominance	0.071	-0.148	0.196	0.008
Facial maturity	0.127	-0.118	0.174	-0.027
Likeability	-0.368^∗^	0.298	-0.231	0.231
Trustworthiness	-0.169	0.284	-0.112	0.238
Youthfulness	-0.124	0.234	-0.235	0.240
Provincial Appearance	0.203	-0.474^∗∗^	0.251	-0.154

^∗^*p < 0.05, ^∗∗^*p <* 0.01, ^∗∗∗^*p* < 0.001*.

## Experiment 3: Double Categorization Task

The rating results of Experiment 2 and the correlational analyses above provided some evidence suggesting that the political membership of candidates’ could be speculated by the perceived provincial appearance of their headshot photos. However, the analyses were based on correlations obtained from two different groups of participants and two different task formats (paper-and-pencil for rating and computerized task for categorization). The evidence was not direct. Thus, Experiment 3 aimed to explore the possible correlation between categorization accuracy of political parties and the particular trait “provincial appearance” from young adults more directly using a within-subject design. We conducted a double categorization task for “political membership” and the trait *“provincial appearance.”* The participants’ task was to categorize candidates as either DPP or KMT members and categorize the same candidates as “Mainlander/Extra-Provincial” or “Native Taiwanese” in two separate sessions.

### Participants

In Experiment 3, we recruited a total of 37 (24 females) non-politically affiliated undergraduates. The participants’ age was between 18 and 25. The sample size was determined before any data analysis and the number of participants was chosen to comply with the study from Experiment 1. Participants were primarily students at China Medical University, Taichung, Taiwan. Written informed consent was obtained before the experiment. All participants self-reported with a normal or corrected-to-normal vision (20/20) and were naïve to the purposes of the experiment. Each participant was tested individually in a quiet, moderately lit room. After completing the task, participants received a cash payment for their participation. One participant was later excluded because he was able to correctly recognize a large number of candidates (30 out of the 80 candidates). The final data set consisted of 36 participants (24 females) with an average age of 22.54 years old (*SD* = 1.56 years).

### Stimuli and Procedure

Experiment 3 was adopted from Experiment 1, however, in addition to asking participants whether the candidate is DPP or KMT in the *“Membership Categorization session,”* we also ask the participant if the candidate is “Mainlander” or “Native Taiwanese” in the *“Provincial Appearance Categorization session.”* The test order of the two sessions was counter-balanced among participants. The stimuli of each session were identical; each consists of 80 photos (40 from each party) from Experiment 1 that were divided into 4 blocks (20 photos per block) by their perceived age, with a ratio of male to female candidates of 4:1.

The procedure of *“Membership Categorization session*” was identical to the task in Experiment 1. As shown in **Figure 2A**, each trial began with a fixation cross for 1.5 s, followed by a photo of either a DPP or a KMT candidate presented for 1.5 s, and the participants were prompted with two subsequent questions without time limit: “Which political party do you think he/she belongs to, KMT or DPP?” and “Do you already know his/her political party?” When participants answered the first question, the second question appeared on the screen and remained on until the participants made a yes/no response. Then the next trial began. The average number of trials of the candidates that the participants reported to have known their political party was 1.90 (the median was 1). These trials were later excluded in the subsequent analyses.

For the *“Provincial Appearance Categorization session*,” the procedure was similar to that of the *“Membership Categorization session*,” which is shown in **Figure 2B**. However, the two subsequent questions were “Which provincial group do you think he/she belongs to, Mainlander or Native Taiwanese?” and “Do you already know this person?” If the participants have any of the background knowledge of the candidates (e.g., the homeland or region, the company or association related to the candidates) means they “know” this person. The average number of trials of the candidates that the participants reported to have known this person was 2.03 (the median was 0). These trials were excluded in the subsequent data analyses.

### Results

**FIGURE 5:**
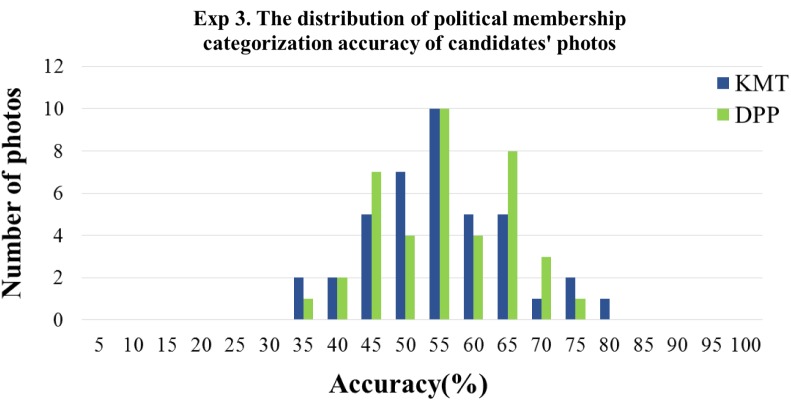
The distribution of item difficulties in Experiment 3, measured by the percentage correct collapsed across participants. The abscissa denotes the categorization accuracy of the candidates’ photos in 5% bins; the ordinate denotes the number of photos.

The primary goal of Experiment 3 was to examine whether participants could categorize the political membership of KMT and DPP candidates by their photos better than chance. At the same time, we also examined whether they categorize the candidate’s provincial appearance as either Mainlanders or Native Taiwanese in a non-random fashion. Thus, we first compared the political membership and provincial appearance categorization accuracy with chance level (0.5) by one-sample *t*-tests. For the political membership categorization task, the results showed that the mean accuracy (*M* = 0.526, *SD* = 0.063) was significantly higher than the chance level [*t*(35) = 2.459, *p* = 0.019, Cohen’s *d* = 0.858]. The result basically replicated the results from Experiment 1. We also analyzed the sensitivity index d prime (d’) and the response bias *c* based on the Signal Detection Theory. The hit rate for KMT was 0.528, the false alarm rate 0.474, yielding a *d’* = 0.135 (*SD* = 0.329). The response bias *c* was.003 (*SD* = 0.037), indicating a very slight bias toward categorizing photos as KMT. The categorization accuracy between the KMT (*M* = 0.528, *SD* = 0.065) and the DPP (*M* = 0.526, *SD* = 0.067) candidates’ photos was not significantly different *(p* = 0.311). To ensure that the above results were not due to a few extremely easily categorized photos that biased the overall accuracy upward (or extremely difficult items that pulled the accuracy downward), we further analyzed the distribution of item difficulties. The difficulty of the photos was defined as the correct percentage of each trial. As shown in **Figure 5**, the correct percentage of candidates’ photos were between 35 to 80%, none of the accuracies of the photos reached 100 or 0%, meaning that the aforementioned greater-than-chance performance was not due to just a few photos that could be easily categorized.

**FIGURE 6:**
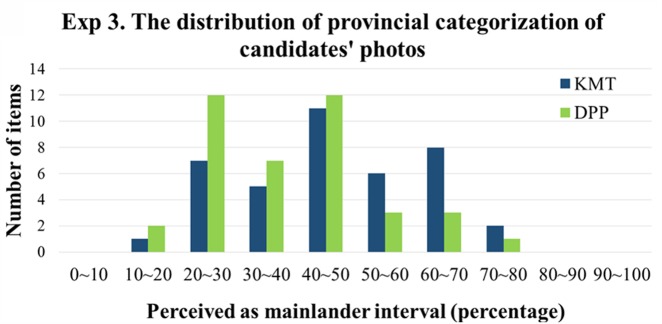
The distribution of provincial appearance categorization in Experiment 3, measured by the percentage of perceived as mainlander collapsed across participants. The abscissa denotes the *provincial appearance* categorization tendency as mainlanders of the candidates’ photos in 10% bins the ordinate denotes the number of photos. The item that falls above 50% means that more than half of the participants perceive the photo as resembling mainlander. The item that falls below 50% means that more than half of the participants perceive the photo as resembling native Taiwanese.

For the provincial appearance categorization task, the results showed that the participants’ categorization of the perceived provincial appearance of KMT and DPP candidates deviated from chance level (0.5). They tended to rate real KMT candidates as Mainlanders [*M* = 0.541, *SD* = 0.082; *t*(35) = 2.992, *p* = 0.005, Cohen’s *d* = 1.011] and to rate the real DPP candidates as the native Taiwanese [*M* = 0.533, *SD* = 0.049; *t*(35) = 4.065, *p* < 0.001, Cohen’s *d* = 1.374]. Likewise, to ensure that the above results were not due to a few extremely easily categorized as either Mainlanders or Native Taiwanese that biased the overall tendency, we further analyzed the distribution of the categorization tendency. **Figure 6** illustrates the distribution of the provincial appearance categorization of the 80 photos. The categorization tendency of the photos was defined as the percentage of being categorized as Mainlanders of that particular trial across participants. 100% means that all participants categorized the photo as a mainlander, 0% means that all participants categorized the photo as Native Taiwanese, and 50% means that half of the participants categorized as mainlander while the other half categorized as Native Taiwanese. As shown in **Figure 6**, the range of the percentage of categorized as Mainlanders of candidates’ photos was from 10 to 80%, none of the percentages reached 100 or 0%, meaning that the aforementioned greater-than-chance performance was not due to just a few photos that could be easily categorized.

**FIGURE 7:**
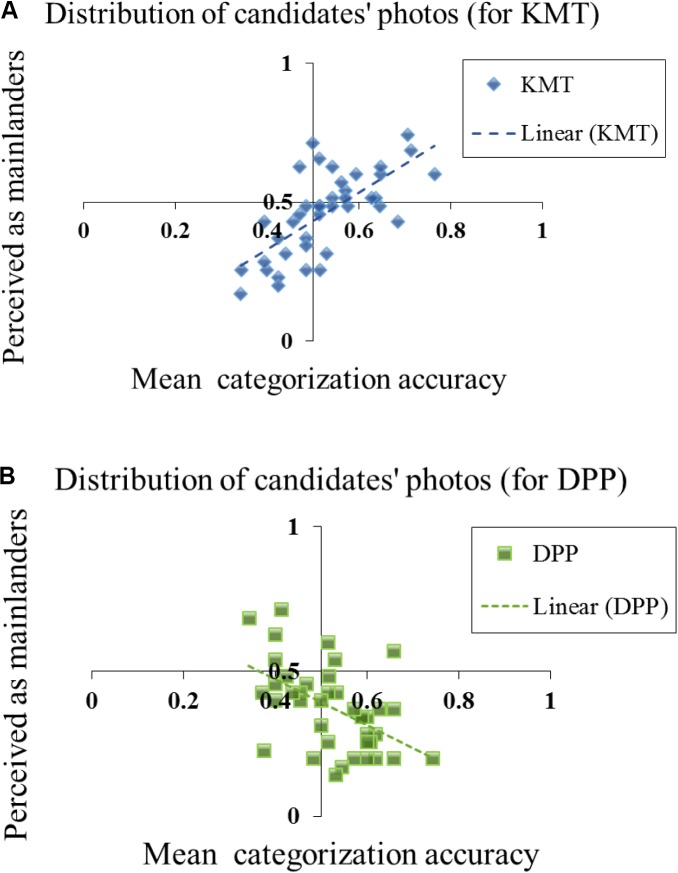
The scatter plots of the accuracy of political membership categorization task (*x*-axis) as a function of *provincial appearance* categorization tendencies (*y*-axis) as mainlanders for each candidate’s photo from KMT **(A)** and DPP members **(B)**. The abscissa denotes the accuracy of categorization. The ordinate represents the percentage of perceived as mainlanders. A candidate’s photo falls above 0.5 means that more than half of the participants perceive the photo as resembling mainlander. A photo falls below 0.5 means that more than half of the participants perceive the photo as resembling native Taiwanese.

To further explore the possible correlation between the accuracy of membership categorization task and how the KMT and DPP candidates are being perceived as Mainlanders or native Taiwanese, we performed two correlation analyses for the KMT and DPP candidates’ photos separately. As shown in the scatter plots of **Figure 7**, we conducted two separate analyses for the KMT and DPP candidates’ photos using the same coordinates. The abscissa denotes the accuracy of categorization (from 0 to 1); the ordinate represents the percentage of perceived as mainlanders (i.e., A candidate’s photo falls above 0.5 means that more than half of the participants perceive the photo as resembling mainlander. A photo falls below 0.5 means that more than half of the participants perceive the photo as resembling native Taiwanese). As shown in the **Figure 7A**, the KMT candidates’ photos exhibited a significant positive correlation between the accuracy of party’s membership categorization task and the perceived provincial appearance score as mainlanders (*r* = 0.690, *p* < 0.001). In addition, most of the data points fall in the first quadrant (i.e., the photos that were tended to categorized as mainlanders and their accuracies were greater than 0.5) and the third quadrant (i.e., the photos that were tended to categorized as native Taiwanese and their accuracies were less than 0.5). On the other hand, the DPP candidates’ photos (**Figure 7B**) exhibited a significant negative correlation between the accuracy of party’s membership categorization task and the perceived provincial appearance score as mainlanders (*r* = -0.521, *p* = 0.001). In addition, most of the data points fall in the fourth quadrant (i.e., the photos that were tended to categorized as native Taiwanese and their accuracies were greater than 0.5) and the second quadrant (i.e., the photos that were tended to categorized as mainlanders and their accuracies were less than 0.5). In another word, the results provide further evidence that that the KMT and DPP candidates’ political membership was strongly associated with the perceived provincial appearance of their headshot photos.

## Discussion

We extended the study from Hu et al. (2016) and discovered that there was indeed a difference in perceived age between KMT and DPP candidates (pilot study), where the KMT candidates were perceived slightly older (peak at 45–50-year-old) than the DPP candidates (peak at 40–45-year-old). We then adjusted these two age intervals to minimize the perceived age difference between KMT and DPP (including male and female candidates between both parties). Candidates from both parties were then equally divided into 4-blocks by their perceived age range to minimize the potential bias on categorization. We found that young participants could categorize KMT and DPP members by their photos better-than-chance even when the perceived age difference has been eliminated (Experiment 1), which supports Hu et al. (2016)’s finding. Furthermore, we discovered the trait that is critical for differentiating between KMT and DPP for both age groups (young adults and middle-aged adults) is “provincial appearance” (Experiment 2). In addition, the middle-aged adults rated marginally higher in “Facial Maturity” for KMT candidates; in another word, KMT candidates’ photos appeared more mature than DPP. Lastly, young adults are likely to use the trait “provincial appearance” as the main characteristic cues to categorize candidates’ political membership (Experiment 3).

One major finding of the present study was that “provincial appearance” is the core trait differentiating the two parties. In the trait rating task (Experiment 2), given that the participants had no information about the candidates’ political affiliation, the KMT candidates’ photos were rated to a resemblance toward mainlanders, and DPP candidates were rated to a resemblance toward the native Taiwanese. This rating tendency was observed in both younger and middle age adults, but the difference in the provincial appearance rating between KMT and DPP candidates was signfincantly greater among middle age adults. Furthermore, in the double categorization task (Experiment 3), the within-subject correlations between the accuracy of membership categorization and the responses on provincial appearance categorization were far from random. The trait “provincial appearance” appeared to be used by young adults to speculate candidates’ political parties. If candidate A’s photo resembles a mainlander, this person was also more likely to be categorized as KMT. On the other hand, if candidate B’s photo resembles native Taiwanese then this person was more likely to be categorized as DPP. What’s also worth noting was that the strong association between the provincial appearance characteristics (mainlander vs. native Taiwanese) and political membership (KMT vs. DPP) was revealed by the errors as well. If one considered a KMT candidate’s photo as resembling native Taiwanese, then this candidate was likely to be categorized as DPP (**Figure 7A**: the data points on the bottom-left quadrant).

To explain this result, we may affiliate “provincial appearance” with the history and development of the two parties in Taiwan. Around 85% of all Taiwanese are descendants from Chinese who came to the island from the Southern Fujian province around 1680. In Taiwan, the term native Taiwanese is usually reserved for people who lived on the island before 1949; those that arrived after are referred to as mainlanders or nationalists (Hays, 2015). Nationalists were also known as the Chinese Nationalist Party or Kuomintang (KMT). The term provincial appearance was typically used to distinguish native Taiwanese from those that arrived from mainland China. Hence the word provincial is typically defined as “belonging to some particular province,” whereas most current KMT candidates are descendants of former KMT troops that came to Taiwan after 1949. The first opposition party in Taiwan, known as the DPP was established in 1986 as the first Taiwanese-born political party in Taiwan, and the majority of the candidates are native Taiwanese. As to the finding from our Pilot Study, where KMT candidates are perceived older in age, and in comparison, KMT has been in Taiwan since 1949. Thus their image could also be stated as being more mature.

Relating to Rule and Ambady’s (2010) study, they demonstrated that North American university students could differentiate Democrats from Republicans, and such categorization was achieved via spontaneous trait-inference (Willis and Todorov, 2006) relating to the party’s images. For young adults in the United States, the faces of Democrats were perceived to be warmer (being rated higher on likeability and trustworthiness), while the faces of Republicans were perceived to be more powerful (being rated higher on dominance and facial maturity). In the present study, we anticipated that KMT’s image is closer to Republicans whereas the DPP’s image is closer to Democrats. In other words, KMT’s image shall be perceived more “powerful” and DPP’s image shall be “warmer.” In middle-aged adults, indeed, “Facial Maturity,” an important trait representing “Power,” is rated significantly higher for KMT candidates, which partially supported our original hypothesis that we anticipated KMT to be perceived as more powerful. However, for the young adults, the rating scores for first four traits (dominance, facial maturity, likeability and trustworthiness) did not differ between KMT and DPP candidates, suggesting that neither “warmth” nor “power” is representative to portray the images of KMT and DPP candidates in the eyes of Taiwanese young generation.

One possible explanation for why Taiwanese young adults did not differentiate KMT and DPP candidates by any trait relating to “Power” or “Warmth” may be due to a lack of civic or political engagement. According to the Social Indicators 2012 (Directorate General of Budget, Accounting, and Statistics [DGBAS] of Executive Yuan, Republic of China, 2013), only 20% young civilians had participated at least one political activity such as presidential campaign rally, or attend a political meeting on public affairs (15–24 years old: 19%, 25–34 years old: 20%). Compare to the situation in the United States, Smith et al. (2009) reported that about 60% young civilians had participated at least one civic or political activities in 2008 (18–24 years old interval: 59%, 25–34 years old interval: 62%). This discrepancy in civic or political engagement may lead to the differential empowerment of the political parties’ images. The stereotypical images of the Democrats and Republican may have a stronger influence on young adults in the United States, whereas the stereotypical images of the KMT and DPP may have less influence on young adults in Taiwan.

In addition to the aforementioned age differences, the present study also discovered that young and middle-aged participants might “read out” different traits manifested on the KMT and DPP candidates’ facial appearances. Franklin and Zebrowitz (2016) reported that the preditors for the young and older adults’ voting behavior differed. In their study, ratings of attractiveness, competence, and trustworthiness of the candidates could positively predict both young and old adults’ voting performance, but the predictability of “competence” was rather weak for older adults. Moreover, “babyfaceness” was negatively associated with the voting performance only for older adults. In another study, Berggren et al. (2017) tested participants from Europe, US, and Australia and found facial attractiveness is associated with higher chances of being conservative and rewarded with votes. Although we did not directly measure the voting preferences in the present study, we did observe a similar age effect as reported by Franklin and Zebrowitz (2016) —the middle-aged adults tended to focus more on “Facial Maturity” of the candidates, which was also an important trait for middle-aged adults to differentiate between KMT and DPP candidates. However, such reliance on facial maturity was not apparent for the Taiwanese young adults.

Broadly speaking, the main findings of the present study complement the results of Hu et al. (2016) in two ways. First, the adult’s performance in the categorization task (Experiment 1) was consistent with Hu et al. (2016), even though the perceived age difference between KMT and DPP candidates’ photos has been minimized, suggesting that the accuracy in categorization observed in Hu et al. (2016) may not be attributable to the perceived age difference between KMT and DPP candidates’ photos. Second, in performing the political membership categorization task, Hu et al. (2016) revealed that the “good guessers” adopted face-to-trait inference as their strategy more often than those participants who were the average guessers, however, this observation was primarily based on self-report. In the present study, we directly asked the young and middle-aged participants to rate the candidates on six target traits and discovered that in both groups, the KMT candidates were perceived as resembling the mainlanders while the DPP candidates were perceived as resembling the native Taiwanese. Despite the difference in methodology between the two studies, our results provide a possible explanation for the “good guessers” in Hu et al. (2016)–They might have adopted “provincial appearance” as a key component or strategy to make face-to-trait inference in the political membership categorization task.

## Conclusion and Future Work

The first impressions of a political candidate’s headshot photo might play an essential role when one evaluates the candidate. In the same line of research, the present study complements the results of Hu et al. (2016) and supports the generality of Rule and Ambady’s (2010) finding in East Asia. When the perceived age difference between the two major Taiwanese political parties, DPP and KMT, was minimized, we demonstrated that adults can still categorize KMT and DPP candidates by their head-shot photos significantly more accurate than chance. Additionally, “provincial appearance” is the most reliable trait to differentiate the two parties for both young and middle-aged adult participants. Last but not least, “provincial appearance” is not only the most reliable trait to differentiate the two parties for both young and middle-aged adult participants, but it is also likely to be used as a major cue to categorize between the two parties among Taiwanese young adults. Although both generations used “provincial appearance” as a major cue, however, the young adults’ and middle age adults’ representations on these political candidates might reflect some changes of the meaning of the term “provincial” over the past 20 years.

Some limitations are noticed upon the study. First of all, although we observed better-than-chance results in the categorization accuracy, but the effect size was relatively small. This could be due to individual differences among the participants, but we did not further explore the impact of individual differences in the present study. Second, participants from different regions (northern or southern Taiwan) would possibly be biased when rating the six target traits (dominance, likeability, etc.) for different candidates. As we observed, participants from the central and northern Taiwan regions may be favoring the KMT party, thus may rate them with a more positive trait, and rate the opposed party (DPP) with a more negative trait. On the other hand, participants from the southern Taiwan may prefer the DPP party, thus may rate them with a more positive trait, and rate the KMT candidate with a more negative trait. However, over-interpretations of these results are another limitation that should be aware of. In the future, we would like to enrich the representativeness of the sample from nationwide (i.e., draw participants from different regions of Taiwan) by using web-based online surveys.

## Ethics Statement

The present study adhered to the guidelines suggested by the Institution Review Board of China Medical University Hospital Research Ethics Committee, Taichung, Taiwan. The participants were informed of the general purpose and the experimental procedures of the study. Written consent of the participants was obtained before the experiment. After finishing the experiment, the participants received a cash payment for their participation.

## Author Contributions

C-KC and SC developed the study concept. C-KC performed the testing and data collections. MH and C-KC performed the data analysis, graphing, and interpretation under the supervision of SC. SC provided critical revisions and editing. All authors contributed to the study design, wrote the manuscript, and approved the final version of the manuscript for submission.

## Conflict of Interest Statement

The authors declare that the research was conducted in the absence of any commercial or financial relationships that could be construed as a potential conflict of interest.

## Footnotes

1 https://goo.gl/yyQh9B
